# Triptorelin for the treatment of adenomyosis: A multicenter observational study of 465 women in Russia

**DOI:** 10.1002/ijgo.13341

**Published:** 2020-09-19

**Authors:** Elena Andreeva, Yulia Absatarova

**Affiliations:** ^1^ Endocrinology Research Centre Moscow Russia; ^2^ Department of Reproductive Medicine and Surgery Evdokimov Moscow State University of Medicine and Dentistry Moscow Russia

**Keywords:** Adenomyosis, Dysfunctional uterine bleeding, Dysmenorrhea, Endometriosis, Fertility, Pain, Pregnancy, Triptorelin

## Abstract

**Objective:**

To evaluate the effectiveness of triptorelin for the treatment of adenomyosis, the benign invasion of endometrial tissue into the myometrium, as a fertility‐preserving alternative to the gold standard hysterectomy.

**Methods:**

In this multicenter, open‐label, observational study in Russia, performed from November 3, 2011, to August 24, 2015, we assessed the efficacy and safety of triptorelin 3.75 mg administered intramuscularly every 28 days in Russian women who were gonadotropin‐releasing hormone agonist treatment‐naïve, aged 25–40 years, and had a diagnosis of endometriosis or adenomyosis with heavy menstrual bleeding. We performed a medical record review, interviews to assess symptom severity, and pelvic assessments including transvaginal ultrasound. Data were obtained at first injection of triptorelin (visit 1), on the day of last injection (visit 2), 6 months after last injection (visit 3), and 9 months after last injection (visit 4). Significance was assessed by Wilcoxon signed rank test.

**Results:**

A total of 465 women were included. There was a significant improvement from baseline in severity of heavy menstrual bleeding in 390/463 (84.2%) of women 6 months after last injection (*P*<0.0001). Severity of dysmenorrhea, abnormal uterine bleeding, and pelvic pain was decreased at visit 3 compared with baseline (*P*<0.0001). Endometriosis symptoms stopped in 253/262 (96.6%) of women at visit 2 and in 243/263 (92.4%) of women at visit 3. Pregnancy was reported in 116/465 (24.9%) women within 9 months following the end of treatment.

**Conclusion:**

Triptorelin has a favorable safety profile, is highly efficacious in treating clinical symptoms of adenomyosis, and improves reproductive function.

ClinicalTrials.gov registration number: A‐38‐52014‐191, registered October 2011.

## 1 INTRODUCTION

Endometriosis is a common gynecologic disease occurring worldwide in approximately 10% of women of childbearing potential, 38%–50% of infertile women, and almost 87% of women with chronic pelvic pain.^1^, ^2^, ^3^, ^4^

Adenomyosis is a form of endometriosis that is characterized by benign endometrial invasion into the myometrium, leading to diffuse enlargement of the uterus,^5^, ^6^ which manifests with ectopic endometrial glands and stroma surrounded by hypertrophic and hyperplastic myometrium. The prevalence of adenomyosis at hysterectomy ranges from 5% to 70% of women.^5^ Symptoms of adenomyosis include menstrual disorders and persisting pain syndrome, which can reduce quality of life.^7^ Adenomyosis can also impair reproductive function, resulting in infertility, by affecting uterotubal transport and altering endometrial function and receptivity. In 50% of cases, adenomyosis can reduce the likelihood of pregnancy occurring naturally. Women with adenomyosis have poor reproductive outcomes compared with those without adenomyosis.^8^ Limited data are available concerning the efficacy of different treatment options in improving fertility.^9^ The most common comorbidity of adenomyosis is uterine fibroids (80%–85% of cases), which may result from similar pathogenic mechanisms.^10^

Hysterectomy is used worldwide to treat adenomyosis in premenopausal and perimenopausal women with severe clinical symptoms^4^, ^6^, ^9^; however, this major surgery can cause complications, reduce quality of life, and incur high economic costs.^5^ Adenomyosis is more prevalent among women of childbearing potential than previously thought,^5^ and alternative treatment options are required to preserve reproductive function.^4^

Gonadotropin‐releasing hormone (GnRH) agonists are used in nonsurgical therapy for all forms of endometriosis, as both main and adjuvant therapy. This treatment reduces both the intensity of endometriosis symptoms and uterine volume.^10^, ^11^, ^12^

Triptorelin acetate 3.75 mg, a 28‐day, prolonged‐release GnRH agonist approved for endometriosis treatment,^13^ has been shown to decrease endometriosis symptom severity and improve reproductive function.^3^, ^14^, ^15^, ^16^, ^17^ However, no large‐scale clinical trials of triptorelin have been performed in women with adenomyosis. We conducted a large, multicenter, observational study to evaluate the effectiveness of triptorelin to treat adenomyosis in routine clinical practice.

## 2 MATERIALS AND METHODS

A national, multicenter, open‐label, observational, non‐interventional study (ClinicalTrials.gov number A‐38‐52014‐191) was performed between November 3, 2011, and August 24, 2015, at 33 study sites in the Russian Federation. The study included GnRH agonist treatment‐naïve women aged 25–40 years with adenomyosis and heavy menstrual bleeding (HMB). Diagnosis was based on uterine enlargement observed during bimanual pelvic and transvaginal ultrasound (TVUS) examinations, performed within 2 months before the first study‐drug injection. Endometriosis disease severity (stage I, II, or III) was categorized using criteria routinely employed in Russia for evaluation of adenomyosis (Table S1).^18^ To avoid recruitment bias, investigators recruited all consecutive patients meeting the inclusion criteria to the study.

Study exclusion criteria included pregnancy, previous treatment with or hypersensitivity to GnRH analogs, and participation in another clinical study (at enrollment and within the last 30 days). The decision to prescribe triptorelin was at the investigator’s discretion and was made before and independently of the decision to enroll a patient. The study was conducted in accordance with the ethical principles of the Declaration of Helsinki.^19^, ^20^ The study was approved by an Independent Interdisciplinary Committee on Ethical Review of Clinical Studies and all patients provided written, informed consent before the study began.

Triptorelin acetate 3.75 mg (Diphereline [Ferring Holding SA, Saint‐Prex, Switzerland]) was administered intramuscularly every 28 days for up to 6 months, in line with prescribing information and clinical practice standards.^13^

Data were collected by the investigators at each study site at four study visits. At visit 1, baseline data were collected on‐site on the day of the first triptorelin injection. Follow‐up data were obtained at: visit 2 (on‐site), last triptorelin injection; visit 3 (on‐site/telephone interview), 6 months after last injection; and visit 4 (telephone interview), 9 months after last injection. Accurate data reporting and protocol compliance were monitored by a data monitor. Data management and statistical analysis were conducted by a contracted clinical research organization (CRO) in accordance with sponsor and CRO standard operating procedures.

At visits 1 and 2, endometriosis symptom intensity (pelvic pain, dysmenorrhea, HMB, and abnormal uterine bleeding [AUB]) was assessed according to the PALM‐COEIN (polyp; adenomyosis; leiomyoma; malignancy and hyperplasia; coagulopathy; ovulatory dysfunction; endometrial; iatrogenic; and not yet classified) classification system, monitored during the previous month by the patient using a four‐grade scale: none, mild, moderate, or severe.^21^ Bimanual pelvic and TVUS examinations were performed at visits 1 and 2. During visit 1, results were collected from TVUS performed within 2 months before first injection (TVUS was conducted during the first 5–10 days of the menstrual cycle). At visit 3, intensity of endometriosis symptoms was assessed and cases of clinical pregnancy or surgical treatment of endometriosis were recorded; at visit 4, only cases of pregnancy or surgical treatment of endometriosis were recorded.

The primary study objective was evaluation of triptorelin effectiveness, assessed by reduction in HMB intensity 6 months after last injection (visit 3). Patients with a decrease in HMB intensity by at least one grade were considered treatment responders. The secondary objectives were to determine: endometriosis symptoms intensity by disease stage (I, II, or III) at end of treatment (visit 2) and 6 months after last injection (visit 3); uterine volume by endometriosis disease stage at end of treatment; and reproductive function 6 and 9 months after last injection by endometriosis disease stage. The change in intensity of each endometriosis symptom was calculated separately; complete treatment response was defined as a decrease in intensity of all endometriosis symptoms by at least one grade. Additional, equivalent analyses were conducted for patients with adenomyosis and uterine myoma.

Anteroposterior uterine size and uterine volume were assessed by TVUS (visits 1 and 2). Uterine volume (cm^3^) was calculated using the formula 0.5230 × *a* × *b* × AP, where *a* and *b* are uterine length and width, respectively, and *AP* is anteroposterior uterine size.^22^ Reproductive function was assessed by the number of recorded clinical pregnancies.

Spontaneously occurring safety events were recorded according to the registered drug’s safety procedure; investigators registered and reported to the manufacturer’s pharmacovigilance department any drug‐related adverse events or serious adverse events via a spontaneous adverse event report form. Adverse events considered by the investigator to be unrelated to the study drug were not collected.

Collected data were analyzed using software SAS version 9.2 (SAS Institute, Cary, NC, USA). Data were expressed using descriptive statistics (mean, standard deviation [SD], median, minimal and maximal value, range, and number of valid cases for quantitative variables; and number, percentage, and distribution for qualitative variables). A *P* value less than or equal to 0.05 was considered statistically significant. Two‐sided 95% confidence intervals (CI, Clopper‐Pearson for qualitative variables) were also presented where relevant. The evolution of severity of symptoms compared with baseline conditions was assessed using a Wilcoxon signed rank test. The sample size calculation was based on the assumption that 10% of patients scheduled to receive GnRH treatment had stage I disease. Inclusion of 400 patients would allow an estimate of the proportion of overall responders with a precision of 4.9%, and of stage I patients with a precision of 15.5% (assuming a two‐sided 95% CI). Assuming that 20% of the patients would be non‐evaluable, up to 500 patients were calculated as needing to be enrolled to give sufficient power to the statistical analyses.

Data from the following study populations were assessed: screened set—patients who had completed screening and had given written informed consent; enrolled set—screened patients who had given written informed consent and were included in the study; effectiveness population—all enrolled patients with HMB symptom‐severity data at baseline, who received at least one triptorelin 3.75 mg injection and had at least one post‐baseline assessment of menorrhagia symptom severity; and per protocol population—all patients included in the effectiveness population for whom no major protocol deviations occurred.

## 3 RESULTS

In total, 463 patients (99.6%) completed all study assessments (visits 1–4). Two patients discontinued the study, one after visit 2 and one after visit 3 (Fig. S1). Baseline demographic and disease characteristics are summarized in Table 1. Mean ± SD age at study enrollment was 32.5 ± 4.56 years (range, 22–43 years). The majority of patients were categorized with endometriosis stage II at baseline (246/465 patients [52.9%]) according to Demidov criteria.^18^ During the study, 186 women (40.0%) received four injections, 169 (36.3%) received six injections, and 90 (19.4%) received three injections. Data for one patient were missing at visit 3. The mean ± SD duration of exposure to triptorelin was 101.4 ± 34.74 days (Table S2). In total, 59 women (12.7%) had active myoma at baseline. During the study, 95 women (20.4%) received additional treatment for endometriosis (progestogens, 54/465 [11.6%]; combined oral contraceptives, 39/465 [8.4%]; and other medication, 9/465 [1.9%]).

**Table 1:** Baseline patient and disease characteristics (enrolled set)

	Women who received triptorelin (N=465)
Age, years	
Mean ± SD (range)	32.5 ± 4.56 (22–43)
Age at first diagnosis, years	
Mean ± SD (range)	31.2 ± 4.55 (20–43)
Stage of endometriosis, n (%)^a^	
Stage I	125 (26.9)
Stage II	246 (52.9)
Stage III	94 (20.2)
Age of menarche, years	
Mean ± SD (range)	13.0 ± 1.24 (10–17)
Infertility, n (%)	224 (48.2)
Primary	116 (51.8)
Secondary	108 (48.2)
Proportion of women who have had at least one pregnancy, n (%)	284 (61.1)
Number of pregnancies	
Mean ± SD (range)	2.5 ± 2.09 (1–19)
Concurrent gynecologic disease, n (%)	379 (81.5)
Surgical interventions, n (%)	
Laparoscopy	156 (33.5)
Ovarian cystectomy	78 (16.8)
Ablation of endometriotic nodes	69 (14.8)
Hysteroscopy	50 (10.8)
Dilation and curettage of uterus	48 (10.3)
Myomectomy	36 (7.7)
Previous therapies at baseline, n (%)	109 (23.4)
Oral contraceptives	73 (15.7)
Progestogens	45 (9.7)
Danazol	2 (0.4)
Mifepristone	2 (0.4)

^a^ Effectiveness set.

**Figure 1:**
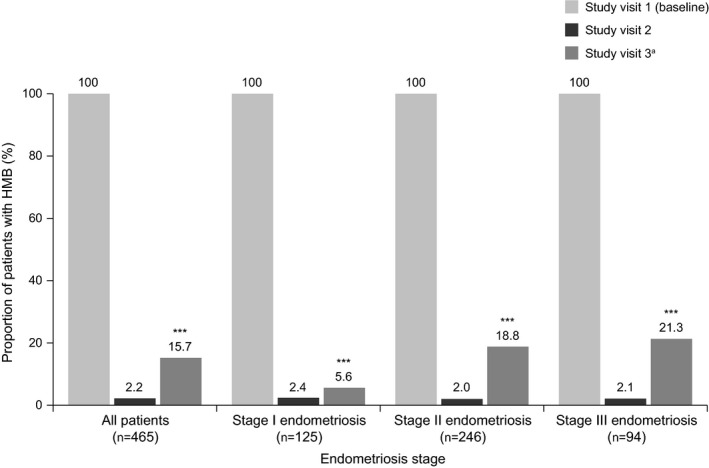
Proportion of women with heavy menstrual bleeding (HMB) during triptorelin treatment, categorized by endometriosis stage (effectiveness set). A statistically significant effect of treatment at visit 3 vs baseline (***P<0.0001) was observed for the overall study group and for each endometriosis stage; significance was calculated using a Wilcoxon signed rank test. ^a^One patient discontinued after visit 2.

At baseline, all patients reported HMB. Treatment with triptorelin eradicated HMB in 390/463 (84.2%) women up to 6 months after the last injection (visit 3; *P*<0.0001; Fig. 1); this effect was observed regardless of endometriosis severity. At visit 2, for the 10 women who reported HMB, intensity was mild (7/465 [1.5%]) or moderate (3/465 [0.6%]). The number of women symptomatic for HMB increased between visits 2 and 3 by 13.5% (visit 2: 10/465 [2.2%], visit 3: 73/463 [15.7%], Fig. 1). At visit 3, the intensity of HMB was mild (59/464 [12.7%]) or moderate (14/464 [3.0%]); no severe cases were reported. Nearly all women responded to treatment (visit 2: 462/465 [99.4%]; visit 3: 453/463 [97.8%]); this was observed for all endometriosis stages (Table 2). For the 59 women with adenomyosis and myoma at baseline, 48/59 (81.4%) patients reported an absence of HMB at visit 3; 9/59 (15.3%) patients had mild symptoms, 2/59 (3.4%) patients had moderate symptoms, and no patients had severe symptoms; 55/59 (93.2%) of these patients had responded to treatment at visit 3 (Table S3).

**Table 2:** Proportion of women who responded to treatment by study outcome and by complete treatment response (effectiveness set)

	Visit 2	Visit 3
Heavy menstrual bleeding^a^		
Overall study population	462/465 (99.4)	453/463 (97.8)
Stage I endometriosis	124/125 (99.2)	124/125 (99.2)
Stage II endometriosis	244/246 (99.2)	239/245 (97.6)
Stage III endometriosis	94/94 (100)	91/94 (96.8)
Patients with adenomyosis and myoma	59/59 (100)	55/59 (93.2)
Dysmenorrhea^a^		
Overall study population	421/423 (99.5)	412/422 (97.6)
Patients with adenomyosis and myoma	56/56 (100)	55/56 (98.2)
Abnormal uterine bleeding^a^		
Overall study population	302/303 (99.7)	296/301 (98.3)
Patients with adenomyosis and myoma	45/45 (100)	44/45 (97.8)
Pelvic pain^b^		
Overall study population	386/398 (97.0)	387/398 (97.2)
Patients with adenomyosis and myoma	56/57 (98.2)	56/57 (98.2)
Complete treatment response^c^		
Overall study population	253/262 (96.6)	243/263 (92.4)
Stage I endometriosis	46/48 (95.8)	43/49 (87.8)
Stage II endometriosis	148/154 (96.1)	144/154 (93.5)
Stage III endometriosis	59/60 (98.3)	56/60 (93.3)
Patients with adenomyosis and myoma	43/44 (97.7)	40/44 (90.9)

Data are presented as n (%).

^a^ Treatment response is defined by a decrease of at least one grade in symptom intensity for those with at least mild intensity symptoms at baseline.

^b^ Patients responded to pain relief.

^c^ Complete treatment response is defined by a decrease of at least one grade in symptom intensity for all symptoms for those with at least mild intensity of symptoms at baseline.

**Figure 2:**
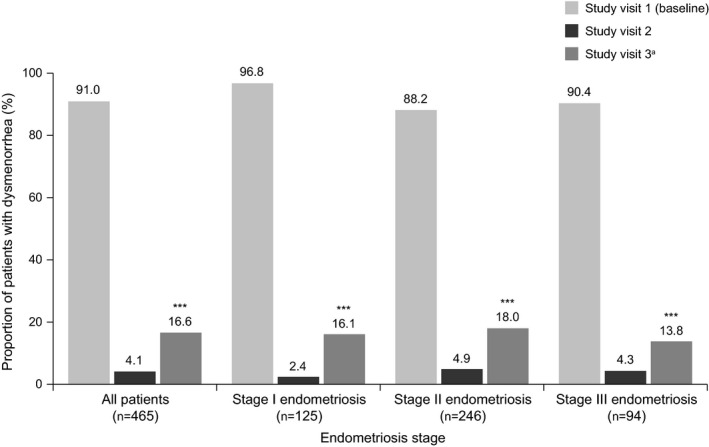
Proportion of women with dysmenorrhea during triptorelin treatment, categorized by endometriosis stage (effectiveness set). A statistically significant effect of treatment at visit 3 vs baseline (***P<0.0001) was observed for the overall study group and for each endometriosis stage; significance was calculated using a Wilcoxon signed rank test. ^a^One patient discontinued after visit 2.

**Figure 3:**
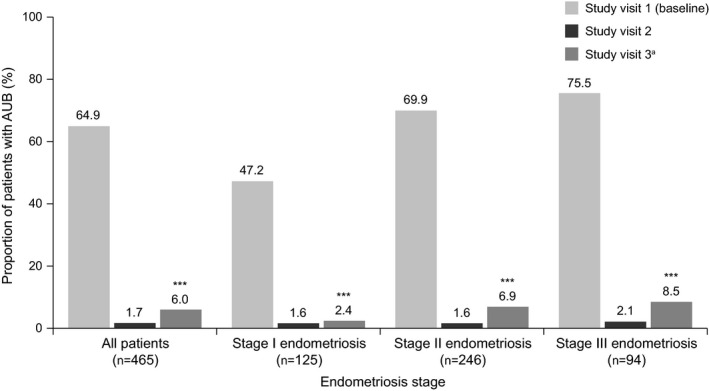
Proportion of women with abnormal uterine bleeding (AUB) during triptorelin treatment, categorized by endometriosis stage (effectiveness set). A statistically significant effect of treatment at visit 3 vs baseline (***P<0.0001) was observed for the overall study group and for each endometriosis stage; significance was calculated using a Wilcoxon signed rank test. ^a^One patient discontinued after visit 2.

**Figure 4:**
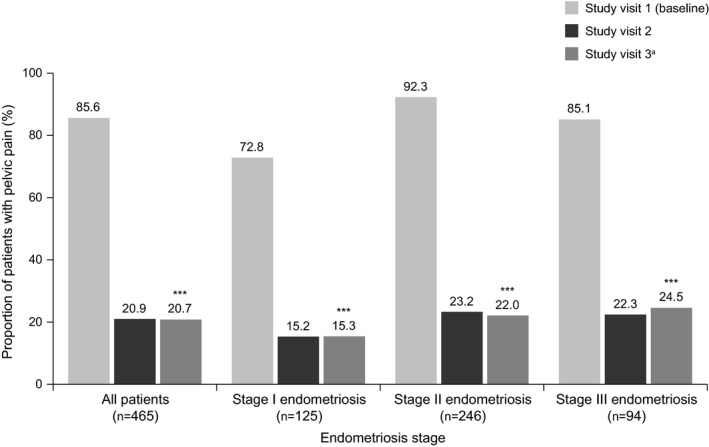
Proportion of women with chronic pelvic pain during triptorelin treatment, categorized by endometriosis stage (effectiveness set). A statistically significant effect of treatment at visit 3 vs baseline (***P<0.0001) was observed for the overall study group and for each endometriosis stage; significance was calculated using a Wilcoxon signed rank test. ^a^One patient discontinued after visit 2.

At baseline, the following proportion of women reported symptoms of: dysmenorrhea, 423/465 (91.0%); AUB, 302/465 (64.9%); and pelvic pain, 398/465 (85.6%; Figs 2, 3, 4). At baseline, pelvic pain was typically moderate (222/465 [47.7%]) or severe (109/465 [23.4%]) in intensity (Table S4).

At visit 3, a significant increase from baseline was observed in the proportion of women without dysmenorrhea symptoms (386/463 [83.4%], *P*<0.0001; Fig. 2), without AUB (435/463 [94.0%], *P*<0.0001; Fig. 3), and without pelvic pain (367/463 [79.3%], *P*<0.0001; Fig. 4). For each of these secondary outcomes, a significant benefit of treatment for each stage of endometriosis was observed at visit 3 (all *P*<0.0001; Figs 2, 3, 4). Similar to the primary outcome, the proportion of patients with symptoms of dysmenorrhea and AUB increased slightly from visit 2 to visit 3 (19/465 [4.1%] vs 77/463 [16.6%], Fig. 2; 8/465 [1.7%] vs 28/463 [6.0%], Fig. 3). The proportions of women without any symptoms of pelvic pain were similar at visits 2 and 3 for the overall population (97/465 [20.9%] vs 96/463 [20.7%], Fig. 4), and at each stage of endometriosis (stage I, 19/125 [15.2%] vs 19/124 [15.3%]; stage II, 57/246 [23.2%] vs 54/245 [22.0%]; stage III, 21/94 [22.3%] vs 23/94 [24.5%], Fig. 4).

At visit 3, a high proportion of women had responded to treatment for dysmenorrhea, 412/422 (97.6%), AUB, 296/301 (98.3%), and pelvic pain, 387/398 (97.2%) (Table 2); findings were numerically similar at study visit 2. Women with adenomyosis and myoma also responded to treatment for each of these secondary outcomes (97.8%–98.2%; Table 2).

A complete treatment response was observed at visit 3 in 92.4% of the overall study population with available data (243/263 patients; Table 2). Similar proportions of patients had a complete treatment response for each stage of endometriosis (stage I, 43/49 [87.8%]; stage II, 144/154 [93.5%]; stage III, 56/60 [93.3%]) and for women with adenomyosis and myoma (40/44 [90.9%]). This treatment pattern was also observed at study visit 2 (Table 2).

By end of treatment (visit 2), the proportion of women with stage II or III endometriosis had significantly decreased from baseline (*P*<0.0001): At baseline, 246/465 (52.9%) had stage II and 94/465 (20.2%) had stage III, whereas at visit 2, 73/376 (19.4%) had stage II and 2/376 (0.5%) had stage III. A total of 89 patients were classified as having “missing data” because they had improvements that corresponded to “no signs of adenomyosis,” which was not an option on the database (Fig. S2).

The changes from baseline in ultrasonic parameters at visit 2 are summarized in Table 3. At visit 2, reductions from baseline in uterine length, width, wall thickness, anteroposterior dimension, and volume were observed (Table 3). Bimanual pelvic examination findings are reported in Table S5, and indicate a beneficial effect of triptorelin treatment on uterine size, shape, mobility, and tenderness.

**Table 3:** Change from baseline in ultrasonic parameters at study visit 2 with triptorelin treatment (effectiveness set)a

	Baseline (visit 1) (N=465)	Visit 2 (N=465)	Change in parameter from baseline
Uterine length, cm	6.1 ± 1.3	5.1 ± 0.8	−1.0 ± 1.1 (−1.1, −0.9)
Uterine width, cm	5.8 ± 1.4	4.8 ± 0.8	−0.9 ± 1.1 (−1.0, −0.8)
Mean uterine wall thickness, cm n	1.0 ± 0.9 410	0.5 ± 0.6 439	−0.5 ± 0.5 406 (−0.6, −0.5)
Anteroposterior size, cm	5.5 ± 1.4	4.5 ± 1.0	−1.0 ± 1.0 (−1.1, 0.9)
Uterine volume, cm^3^	119.3 ± 169.3	62.5 ± 32.7	−56.7 ± 159.3 (−71.2, −42.1)

Abbreviation: SD, standard deviation.

All values are presented as mean ± SD (95% confidence interval).

Pregnancy was reported following end of treatment by 116 of 465 women (24.9%; Table 4); of the 116 pregnancies, 113 (97.4%) were planned, and two abortions took place (0.4%). A total of 32 pregnancies (17.9%) occurred in the 179 women aged 35 years or older (Table 4). In total, 4 of 465 (0.9%) women received a surgical intervention following end of triptorelin treatment; hysterectomy was performed on 1 of 465 (0.2%) women with stage III disease.

**Table 4:** Summary of pregnancy cases following end of triptorelin treatment (enrolled set)

	Any pregnancy since end of treatment	Any pregnancy at 6 months post‐treatment (visit 3)	Any pregnancy at 9 months post‐treatment (visit 4) since visit 3
Overall study, n	465	464	463
All pregnancies	116 (24.9)	57 (12.3)	59 (12.7)
Planned pregnancy	113 (97.4)	55 (96.5)	58 (98.3)
Unplanned pregnancy	3 (2.6)	2 (3.5)	1 (1.7)
Women ≥35 years old, n	179	179	179
All pregnancies	32 (17.9)	16 (8.9)	16 (8.9)
Planned pregnancy	31 (96.9)	16 (100)	15 (93.8)
Unplanned pregnancy	1 (3.1)	0 (0)	1 (6.3)

All data are presented as n (%) unless otherwise stated.

In total, 149 adverse events were reported during the study, the most frequent being hot flush (n=66), followed by irritability (n=10), sleep disorders (n=7), and tearfulness (n=4; Table S6). No serious adverse events were reported during the study.

## 4 DISCUSSION

The results suggest that intramuscular triptorelin acetate 3.75 mg offers an effective treatment for women with adenomyosis and has an acceptable safety profile. The primary study outcome was met, and significant and consistent effects on dysmenorrhea, AUB, and pelvic pain were also reported, regardless of disease stage. Overall, symptoms were improved (complete treatment response) in 96.6% of women at visit 2 and 92.4% of women at visit 3. Of note, triptorelin was effective in patients with concurrent uterine pathology; in patients with myoma, a complete treatment response was observed at visit 2 in 97.7% of women and maintained 6 months after last injection in 90.9% of women (visit 3). Nearly all patients experienced an improvement of endometriosis; by end of treatment (visit 2), only two patients were classified with stage III endometriosis.^18^ Our results confirm other findings showing that triptorelin decreases HMB manifestations and pain in patients with endometriosis, adenomyosis, and uterine myoma.^14^, ^15^, ^16^ This is noteworthy given the high proportion of patients with stage II or III endometriosis, concurrent gynecologic diseases, and different forms of endometriosis in our study population.

During triptorelin treatment, a decrease from baseline in uterine size and volume was observed, which confirms previous findings.^14^, ^15^ Similarly, bimanual pelvic examination revealed reduced uterine size and diminished tenderness at palpation in most patients after treatment. The uterus became softer and returned to a normal (nonspherical) shape, with improved mobility, suggesting reduced adhesive processes in the pelvic cavity.

Adenomyosis treatment options are hormonal therapy, conservative surgery, and hysterectomy. The first drugs licensed for this purpose were GnRH analogs. The pronounced effect of triptorelin (and other GnRH analogs) on the course of endometriosis may be attributed to its capacity to suppress estrogen synthesis, as well as to its ability to inhibit endometrioid foci growth by decreasing the synthesis of anti‐inflammatory cytokines and stimulating apoptosis of ectopic endometrioid cells.^10^ Additionally, GnRH analogs can suppress angiogenesis, decreasing vascular growth factor synthesis.^4^, ^10^, ^23^ Given their multiple effects on the endometrium and myometrium, GnRH analogs could be considered as pathogenic adenomyosis treatments.^23^ This drug class has specific advantages in women with a combination of adenomyosis and uterine myoma. The mechanism of myoma growth inhibition by GnRH analogs is mediated by hypoestrogenic induction, suppression of proliferation (decrease in Ki‐67 expression), increased apoptosis of myoma cells, and significant reduction in expression of all main growth factors and their receptors.^24^, ^25^ The potential of GnRH analogs to induce apoptosis in the endometrium and simultaneously reduce uterine volume, menstrual blood loss, and size of myomatous nodes has been shown in multiple studies.^26^, ^27^

The positive effect of triptorelin on reproductive function is also of interest. Difficulty in becoming pregnant was diagnosed at baseline in almost half of the enrolled patients, in keeping with previous studies^28^, ^29^, ^30^; just under two‐thirds had at least one previous pregnancy before inclusion in the study. After triptorelin treatment, pregnancy was reported in approximately one‐quarter of patients. Markedly, 18% were in women aged 35 years or older, and 20% of all women received contraception as follow‐up treatment. Our data confirm previous results indicating that triptorelin and other GnRH agonists can improve fertility in 20%–25% of women with adenomyosis, independent of stage, within 1 year after treatment completion.^31^

Notably, triptorelin treatment helped to avoid surgical intervention; only one hysterectomy was performed (0.2%), in a woman with endometriosis stage III. Thus, triptorelin may be considered an effective treatment option for women who wish to maintain their reproductive potential. Furthermore, there is growing evidence of long‐term, negative health consequences of hysterectomy compared with natural menopause.^32^, ^33^, ^34^, ^35^

A favorable safety profile of triptorelin was observed during the study treatment period. Only well‐known adverse events were observed, and no serious adverse events were reported. Previously, in a Russian multicenter observational study of triptorelin in patients with endometriosis (N=1000), only 11.3% of patients reported hot flushes during the 4–6‐month study, none of which required additional treatment.^2^

During the current study, 20.4% of patients used additional drugs to treat adenomyosis. Because GnRH agonists cannot be used continuously owing to development of estrogen‐deficiency symptoms,^11^ this raises an important question concerning the need for additional treatments. Hirata et al.^36^ showed that dienogest administration for symptomatic treatment of adenomyosis significantly decreased pelvic pain; however, increased menstrual bleeding was observed in some patients. Additionally, studies have evaluated the potentiation of treatment efficacy and duration after consecutive administration of a GnRH agonist and dienogest (2 mg/d) in women with recurrent endometriosis,^37^ and with combined uterine pathology.^38^ Consecutive treatment in cases of severe endometriosis and/or combined uterine pathology may be a promising treatment approach. There are several study limitations to note. Due to the multicenter study design, there may be some biases based on symptom reporting, and potential variations in ultrasound findings because of minor differences in sensitivity of the ultrasound echo between study sites.

In conclusion, treatment with intramuscular triptorelin acetate 3.75 mg decreased the clinical symptoms of adenomyosis in almost all patients. Considering the favorable safety profile as well as the capacity to improve reproductive function, triptorelin may be considered as a treatment option for adenomyosis, particularly in young women who wish to preserve reproductive potential.

## AUTHOR CONTRIBUTIONS

EA and YA contributed to the study design and implementation. Both authors analyzed and interpreted the study data. Both authors were contributors in writing the manuscript and both authors read and approved the final manuscript.

## CONFLICTS OF INTEREST

The authors have no conflicts of interest.

## Supporting information


**Figure S1**. CONSORT patient flow diagram.

**Figure S2**. Change in endometriosis stage from baseline during triptorelin treatment (effectiveness set). At visit 2, 89 patients had an improvement from stage I endometriosis, but an option for “not applicable/no signs of endometriosis” was not available on the case report form; therefore, these data have not been included.

**Table S1**. Classification of stages of adenomyosis according to Demidov et al.^18^ ^a^
**Table S2**. Patient disposition and treatment exposure.
**Table S3**. Heavy menstrual bleeding (HMB) dynamics during triptorelin treatment in patients with adenomyosis and myoma (effectiveness set).
**Table S4**. Pelvic pain intensity at baseline (effectiveness set).
**Table S5**. Change in pelvic examination parameters during triptorelin treatment (enrolled set).
**Table S6**. Adverse events reported by investigators during the study (enrolled set).
