# Supplementary figures and images for: Triptorelin for the treatment of adenomyosis: A multicenter observational study of 465 women in Russia
Source: Int J Gynaecol Obstet. 2020 Sep 19;151(3):347–54. doi: 10.1002/ijgo.13341 (PMC7756635; doi:10.1002/ijgo.13341)

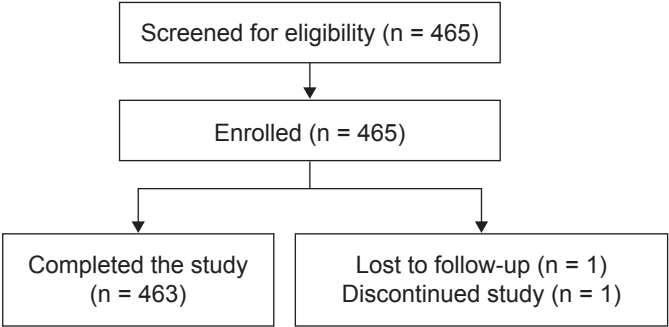

Supplement: Supplementary file 1 — Figure S1. CONSORT patient flow diagram. [file IJGO-151-347-s001.pdf]

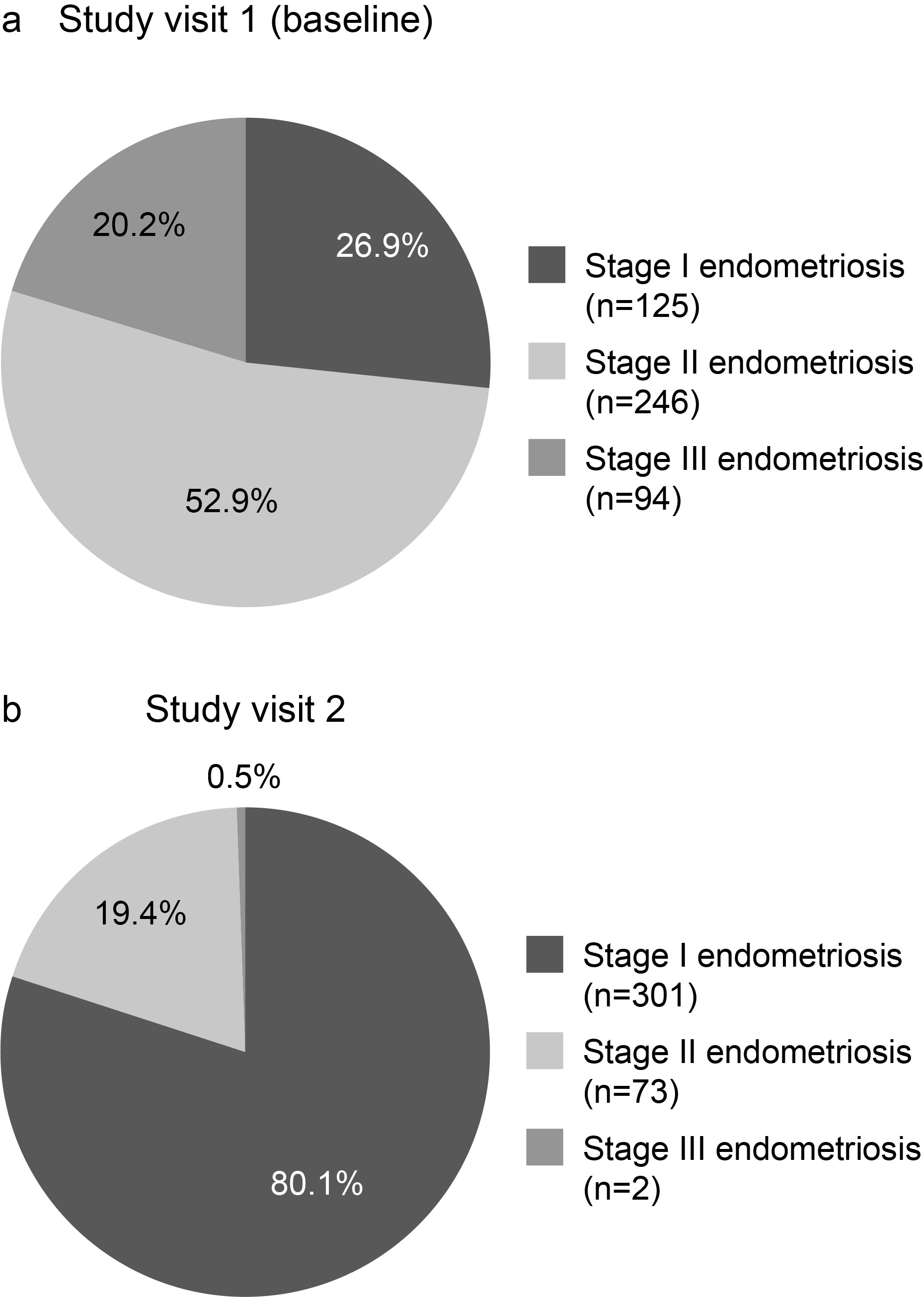

Supplement: Supplementary file 2 — Figure S2. Change in endometriosis stage from baseline during triptorelin treatment (effectiveness set). At visit 2, 89 patients had an improvement from stage I endometriosis, but an option for “not applicable/no signs of endometriosis” was not available on the case report form; therefore, these data have not been included. [file IJGO-151-347-s002.jpg]
