# Supplementary material for: Triptorelin for the treatment of adenomyosis: A multicenter observational study of 465 women in Russia
Source: Int J Gynaecol Obstet. 2020 Sep 19;151(3):347–54. doi: 10.1002/ijgo.13341 (PMC7756635; doi:10.1002/ijgo.13341)
Supplement: Supplementary file 3 — Table S1. Classification of stages of adenomyosis according to Demidov et al. 18 a Table S2. Patient disposition and treatment exposure. Table S3. Heavy menstrual bleeding (HMB) dynamics during triptorelin treatment in patients with adenomyosis and myoma (effectiveness set). Table S4. Pelvic pain intensity at baseline (effectiveness set). Table S5. Change in pelvic examination parameters during triptorelin treatment (enrolled set). Table S6. Adverse events reported by investigators during the study (enrolled set). [file IJGO-151-347-s003.docx]

**Supporting Information**

**SUPPLEMENTARY TABLE S1** Classification of stages of adenomyosis according to Demidov et al. [18].^a^

| **Disease stage** | **Description** |
| --- | --- |
| Stage I endometriosis | - Thickness of uterus is 4.6 ± 0.6 cm - Difference in wall thickness is 0.3 ± 0.2 cm - Hypoechoic zone around the endometrium - Hypo- and anechogenic structures 1–2 mm in the basal layer - Uneven thickness of the basal layer - Serratus or ruggedness of the basal layer - Hyperechoic patches up to 0.3 cm in the myometrium of the basal layer |
| Stage II endometriosis | - Thickness of uterus is 5.1 ± 0.7 cm - Difference in wall thickness is 0.8 ± 0.3 cm - Zone of increased echogenicity is of various thicknesses in the myometrium of the basal layer - Presence, in the zone of increased echogenicity, of anechogenic inclusions of 0.2–1.1 cm, sometimes containing a suspension |
| Stage III endometriosis | - Thickness of uterus is 6.0 ± 1.2 cm - Difference in wall thickness is 2.0 ± 1.2 cm - Hyperechoic zone is more than half the wall thickness in the myometrium - Presence, in the hyperechoic zone, of anechogenic inclusions of 0.2–0.6 cm, sometimes containing a suspension - Sign of vertical bands - Decrease in sound conductivity |

^a^ This classification is routinely used in Russia for the evaluation of endometriosis according to Endometriosis: diagnosis, treatment and rehabilitation. Russian Clinical guidelines. Moscow 2013.

**SUPPLEMENTARY TABLE S2** Patient disposition and treatment exposure.

|  | **Number of patients, n (%)** |
| --- | --- |
| Number of triptorelin injections during the study |  |
| 1 | 1 (0.2) |
| 2 | 3 (0.6) |
| 3 | 90 (19.4) |
| 4 | 186 (40.0) |
| 5 | 14 (3.0) |
| 6 | 169 (36.3) |
| 7 | 1 (0.2) |
| 9 | 1 (0.2) |
| Treatment exposure, days  Mean ± SD  Range  Median (Q1–Q3) | 101.4 ± 34.74 1–228 85.0 (84.0–141.0) |

Abbreviations: Q, quartile; SD, standard deviation.

**SUPPLEMENTARY TABLE S3** Heavy menstrual bleeding (HMB) dynamics during triptorelin treatment in patients with adenomyosis and myoma (effectiveness set).

|  | **Study visit 1 (baseline) (N=59)** | **Study visit 2**  **(N=59)** | **Study visit 3 (N=59)** |
| --- | --- | --- | --- |
| Symptoms of HMB, n (%) |  |  |  |
| None | 0 (0) | 58 (98.3) | 48 (81.4) |
| Mild | 6 (10.2) | 1 (1.7) | 9 (15.3) |
| Moderate | 28 (47.5) | 0 (0) | 2 (3.4) |
| Severe | 25 (42.4) | 0 (0) | 0 (0) |
| Treatment response, n (%) | – | 59 (100) | 55 (93.2) |

**SUPPLEMENTARY TABLE S4** Pelvic pain intensity at baseline (effectiveness set).

| **Pelvic pain intensity** | **Number of patients with pelvic pain, n (%) (N=465)** |
| --- | --- |
| Mild | 67 (14.4) |
| Moderate | 222 (47.7) |
| Severe | 109 (23.4) |

**SUPPLEMENTARY TABLE S5** Change in pelvic examination parameters during triptorelin treatment (enrolled set).

| **Presence of pelvic examination parameter** | **Study visit 1 (baseline) (N=465)** | **Study visit 2 (N=465)** | |
| --- | --- | --- | --- |
|  |  | **Symptom present** | **Symptom absent** |
| Enlarged uterus, n (%)  Yes  No | 448 (96.3) 17 (3.7) | 183 (39.4) 0 (0) | 265 (57.0) 17 (3.7) |
| Tender uterus, n (%)  Yes  No | 318 (68.4) 147 (31.6) | 34 (7.3) 0 (0) | 284 (61.1) 147 (31.6) |
| Dense uterus, n (%)  Yes  No | 367 (78.9) 98 (21.1) | 181 (38.9) 22 (4.7) | 186 (40.0) 76 (16.3) |
| Spherical uterus, n (%)  Yes  No | 434 (93.3) 31 (6.7) | 237 (51.0) 3 (0.6) | 197 (42.4) 28 (6.0) |
| Mobile uterus, n (%)  Yes  No | 317 (68.2) 148 (31.8) | 312 (67.1) 98 (21.1) | 5 (1.1) 50 (10.8) |
| Presence of adhesive processes, n (%)  Yes  No | 178 (38.3) 287 (61.7) | 90 (19.4) 2 (0.4) | 88 (18.9) 285 (61.3) |

**SUPPLEMENTARY TABLE S6** Adverse events reported by investigators during the study (enrolled set).

| **Adverse event** | **n of patients** |
| --- | --- |
| Total | 149 |
| Hot flushes | 66 |
| Irritability | 10 |
| Sleep disorders | 7 |
| Emotional distress | 4 |

**SUPPLEMENTARY FIGURE S1** CONSORT patient flow diagram.

**SUPPLEMENTARY FIGURE S2** Change in endometriosis stage from baseline during triptorelin treatment (effectiveness set). At visit 2, 89 patients had an improvement from stage I endometriosis, but an option for “not applicable/no signs of endometriosis” was not available on the case report form; therefore, these data have not been included.
